# Evaluation of clinical outcomes, complication rate, feasibility, and applicability of transfacet pedicle-sparing approach in thoracic disc herniation: a systematic review and meta-analysis

**DOI:** 10.1186/s13018-023-04016-9

**Published:** 2023-07-20

**Authors:** Shafi Hamid, Farid Moradi, Seyed Reza Bagheri, Mahsa Zarpoosh, Parsa Amirian, Hooman Ghasemi, Ehsan Alimohammadi

**Affiliations:** 1 School of Medicine, Wayne State University, Detroit, MI USA; 2 Department of Neurosurgery, Imam Reza Hospital, Kermanshah University of Medical Sciences, Kermanshah, Iran; 3 Imam Reza Hospital, Kermanshah University of Medical Sciences, Kermanshah, Iran; 4 School of Nursing and Midwifery, Imam Reza Hospital, Kermanshah University of Medical Sciences, Kermanshah, Iran

**Keywords:** Transfacet pedicle-sparing, Thoracic disc herniation, Clinical outcomes, Visual analog scale, Surgical management

## Abstract

**Objective:**

This study aimed to evaluate the clinical outcomes, complication rate, feasibility, and applicability of transfacet pedicle-sparing approach for treating thoracic disc herniation.

**Methods:**

We searched three databases including the Cochrane Library, PubMed, and Embase for eligible studies until Dec 2022. The quality of studies and their risk of bias were assessed using the methodological index for non‐randomized studies. We evaluated the heterogeneity between studies using the *I*^2^ statistic and the *P*-value for the heterogeneity.

**Results:**

A total of 328 patients described in 11 included articles were published from 2009 to 2022. Pain outcomes using the visual analog scale (VAS score) were reported in four studies. The standardized mean difference was reported as 0.749 (CI 95% 0.555–0.943). The obtained result showed the positive effect of the procedure and the improvement of patients' pain after the surgery. Myelopathy outcomes using the Nurick score were reported in five studies. The standardized mean difference was reported as 0.775 (CI 95% 0.479–1.071). The result showed the positive effect of the procedure. Eight studies assessed postoperative complications and neurological deterioration. The pooled overall complication was 12.4% (32/258) and 3.5% (9/258) neurological worsening.

**Conclusion:**

The results of this study demonstrated a positive effect of the transfacet pedicle-sparing approach on the clinical outcomes of patients with thoracic disc herniation surgery. The technique has been shown to be safe and effective for the right patient. The technique is associated with lower rates of complications and a shorter hospital stay compared to other surgical approaches. This information can assist clinicians in making informed decisions when selecting the most appropriate surgical technique for their patients with thoracic disc herniation.

## Introduction

Disc herniation in the thoracic area is uncommon and reported to account for 0.25 to 0.75% of all herniated discs [1]. Historically, given the lack of imaging, thoracic herniation diagnosis relied heavily on the clinical history and physical findings [1, 2]. Previous studies have estimated the incidence of symptomatic disc herniation in the general population to be 1 in thousand to 1 in million [1, 3, 4]. In the second half of the twentieth century, the diagnosis and prognosis of patients with thoracic disc herniation significantly improved because of the development of diagnostic imaging and surgical methods. Radiological studies have shown that thoracic disc herniations occur between 11 and 37% of asymptomatic patients [5–8]. Compared to all herniated discs undergoing surgical procedures, thoracic discectomy accounts for 0.15–4% [9–12].

Thoracic disc herniation was first described by Middleton and Teacher in 1911 after an autopsy was done on a young male patient who initially presented with back pain and inability to walk straight after lifting a heavy weight [13]. TDH is diagnosed more frequently in males than in females, seen often in their 40–50 s [14], mostly located in the lower thoracic spine [15], most frequently occurring between T8 and T12 [15]. This is due to the apparently decreased rigidness in the lower section of the thoracic spine (cite) [16]. Most common disc protrusions are in central or centrolateral locations [1, 10]. The thoracic spine, calcified herniated discs, and giant disc herniations were reported to account for 22–65% and 15%, respectively [17]. Clinical presentation is non-specific. The axial pain and/or radicular pain is often the first presenting symptom [10]. However, in a long-lasting state, myelopathic symptoms may be seen [13, 18, 19]. Symptoms can often mimic other pathologies arising from other neighboring structures within the thorax and the abdomen which can make diagnosis challenging without the aid of imaging [13, 20].

Management is conservative and non-surgical in patients without neurological abnormality and is reported to have a 75% success rate [21]. Thoracic discectomies are reserved for symptomatic patients with signs of myelopathy or refractory radicular pain [1]. In the literature, there is no consensus on which surgical approach is the gold standard, and furthermore, there is a lack of guideline recommendations. Laminectomy and fusion were historically the first procedures to be undertaken to treat TDHs. However, now contraindicated due to major complications like cord ischemia and increased morbidity and death [3, 16, 22, 23]. Due to the smaller cord-to-canal space ratio in the thoracic spine in comparison with this ratio in the cervical and lumbar spine, the posterior approach is not preferable in the thoracic spine [24, 25]. These early experiences led to the development of new surgical techniques to enhance access to the anterior, lateral, and posterolateral views of the thoracic spine [10, 22, 26–31].

Some of the available surgical approaches involve manipulating complex anatomy, resection of the ribs, and violation of the thoracic cavity allowing for potential postoperative respiratory complications and poor outcomes [22, 27, 29, 30]. Several case series have reported positive results utilizing the pedicle-sparing transfacet approach, a posterolateral procedure, initially described by Stillerman et al. as a simpler alternative to the more extensive procedures and report lower postoperative complication rates [10]. The aim of this study was to assess the surgical outcome, complication rate, feasibility, and applicability of transfacet pedicle-sparing approach for treating thoracic disc herniation.

## Methods

We conducted the present study according to the Preferred Reporting Items for Systematic Reviews and Meta-Analyses (PRISMA) statement [32]. No approval from the institutional review board of our hospital was required.

### Search strategy

**Fig. 1:**
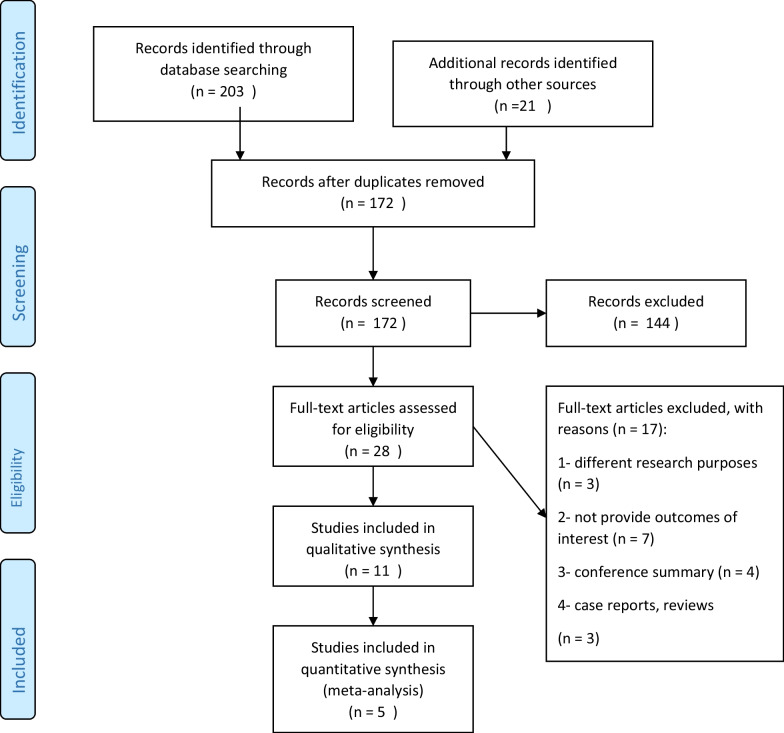
Flowing diagram of included studies selection process

Queried databases included EMBASE, PubMed, and Cochrane. Two authors (PA and MZ) assessed and performed a comprehensive web-based literature search using the following search string (“thoracic disk” OR “disk herniation” OR “diskopathy” AND (“transfacet pedicle sparing” OR “transfacet” OR “pedicle sparing”). No date limitations were applied. Afterward, both authors independently reviewed the titles, abstracts, and full-text studies according to pre-established criteria. Additionally, we queried the bibliographies of included studies for additional relevant articles. Our search flow chart is outlined in Fig. 1. Any disagreement between the two authors was resolved by consensus. Weighted kappa scores were used to assess agreement between the two researchers [33].

### Inclusion and exclusion criteria

To be included, articles had to (1) evaluate transfacet pedicle sparing approach for treating thoracic disc herniation, (2) report results for adult human subjects (≥ 18 years), (3) report patient outcomes, and (4) articles in English. We excluded animal, cadaver, and biomechanical studies, case reports, commentaries, editorials, and reviews.

### Risk of bias assessments and evaluations of validity

Two independent reviewers (PA and MZ) assessed the quality of studies and their risk of bias using the methodological index for non‐randomized studies (MINORS) [33]. We determined the high risk of bias for risk of bias score for non-randomized studies as ≤ 8 (controlled group not present) or ≤ 12 (controlled group present).

### Heterogeneity assessments

We assessed the heterogeneity between studies using the *I*^2^ statistic and the *P*-value for the heterogeneity [34]. We considered *substantial heterogeneity* as ≥ 50% [35].

### Data extraction and outcome assessment

The qualifying full-text publications were systematically searched for several variables that included first author, year of publication, number of patients, gender, age, study design, preoperative assessment, and postoperative assessment as shown in Table 1. The study's main outcome was neurologic recovery, and the secondary outcome was pain relief.

**Table 1:** Characteristics of the eligible studies

							Clinical Presentation				
Authors, year	Country	Study design	# of patients	Number of males/number of females/mean age (years)	Mean Follow-up in months, Range	Loss to F/up	Myelopathy	Radiculopathy	Myelopathy and radiculopathy	Axial back pain	Bladder dysfunction
	US	R	18	11/7/50	12 (4–39)	4					
	US	R/Coh	35	NR/NR/50	8.745	4					
Arnold et al. [51]	US	P	15	6/9/51.1	30	0	67% (10)	100% (15)	67% (10)	67% (10)	
Nishimura et al. [40]	Canada	P	16	6/10/49.5	10 (5–48)	0	94% (15)	88% (14)		25% (3)	31% (5)
Yang et al. [59]	China	P	33	27/6/41.8	37 (12–63)	4					
Carr et al. [37]	US	R	51	20/31/60	(1–46)	1	31% (16)	35% (18)	16% (8)	18% (10)	
Sivakumaran et al. [55]	US	R	24	7/17/56.3	6 (2–36)	0	88% (21)	33% (8)		43% (10)	67% (16)
Çelik et al. [54]	Turkey	P	28	16/12/43.6	33	0					
Kashyap et al. [38]	US	R	86	46/40/NR	12	0					
	Iran	R	19	12/7/46.7	6	0					
Ovalioglu et al. [39]	Turkey	P	11	6/5/53.5	21.02 ( 6–43)	0	73% (8)	64% (7)		10%(10)	55% (6)

R, retrospective; Coh, cohort; P, prospective; US, United States

### Data analysis and statistical analysis

For each study, differences in means, sample size, and *P* value were used to calculate the outcomes (neurological recovery and pain relief). The analysis was performed as 2-tailed. The standardized mean difference was calculated for each study. Heterogeneity was determined using the Cochran *Q* value and the *I*^2^ index. A random effects model was used in cases where heterogeneity was significant, therefore if the *I*^2^ value was greater than 50%. A fixed effect model was used if the I^2^ value was less than 50%.

## Results

### Eligible studies

A total of 328 patients described in 11 included articles were published from 2009 to 2022 and consisted of five articles reported on 214 cases of retrospective case series and 5 articles reported on 103 cases of prospective observational studies undergoing transfacet pedicle-sparing approach. One other retrospective cohort study of 35 cases compared the outcomes and complications between the anterior transthoracic approach and modified transfacet pedicle-sparing (Table 1).

### Patient demographics and characteristics

Four studies reported clinical symptoms and the pooled clinical presentation of these studies was 71% myelopathy, 64% radiculopathy, 42% both myelopathy and radiculopathy, 49% axial back pain, and 51% bladder dysfunction **(**Table 1**)**. Nine studies reported 332 levels of herniation, of which the most common herniated level was T11-12 20% (66), followed by T7–T8 19% (63) and T10–T11 16% (52) **(**Table 2**)**. Six studies reported the position of 176 herniated discs, of which 55% (96) were paracentral and 30% (52) were central, 5% (8) lateral, and 3% (5) were both central and lateral herniation. Calcified discs were 18% (31) of the total 176 herniated discs **(**Table 3**)**. Four studies with a total of 95 patients reported multilevel discs, of which 31 patients had two-level, 4 patients had three-level and 1 patient had four-level disc herniations. Overall, 38% (36/95) of patients had multilevel disc herniations.

**Table 2:** Studies that reported levels of herniated discs

Authors, Year	T1–T2	T2–T3	T3–T4	T4–T5	T5–T6	T6–T7	T7–T8	T8–T9	T9–T10	T10–T11	T11–T12	T12–L1	Total disc herniated
	1	1	0	1	1	2	5	5	3	3	6	1	29
	1	1	0	1	1	5	10	7	6	4	11	0	47
Arnold et al. [51]	0	0	0	0	4	6	10	6	3	1	1	1	32
Nishimura et al. [40]	0	1	0	0	2	1	1	1	1	4	4	1	16
Carr et al. [37]	1	0	1	1	1	10	8	5	5	12	17	5	66
Sivakumaran et al. [55]	0	0	1	0	0	3	7	3	2	6	2	1	25
Kashyap et al. [38]	0	2	3	1	3	10	21	10	5	14	11	6	86
	0	0	0	0	1	0	1	0	2	4	10	1	19
Ovalioglu et al. [39]	0	0	0	0	0	0	0	2	2	4	4	0	12
Total	3	5	5	4	13	37	63	39	29	52	66	16	332
Percentage	1%	2%	2%	1%	4%	11%	19%	12%	9%	16%	20%	5%

**Table 3:** Studies that reported the location of herniated discs

Authors, year	Central	Cetrolateral	Central + lateral	Lateral	Calcified	Total herniated discs
	10	19				29
Nishimura et al. [40]	5	11			8	16
Carr et al. [37]	21	25	5		4	66
Sivakumaran et al. [55]	7	17		1	7	25
Çelik et al. [54]	7	21			6	28
Ovalioglu et al. [39]	2	3		7	6	12
Total	52	96	5	8	31	176
Percentage	30%	55%	3%	5%	18%	

### Neurological and pain outcome

#### Pain

**Fig. 2:**
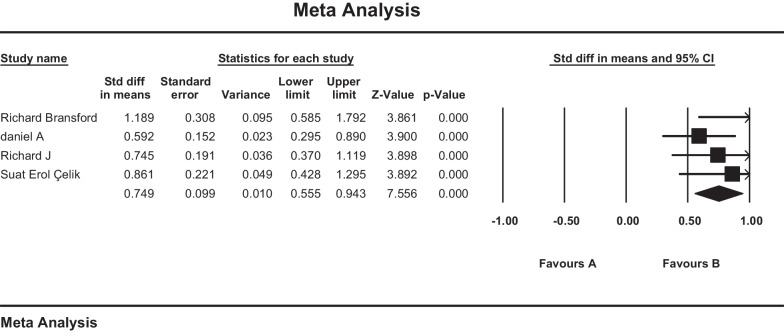
forest plot of standardized mean difference of VAS scale

Pain outcomes using the visual analog scale (VAS score) were reported in four studies in 132 patients undergoing transfacet pedicle-sparing approach for TDH. Due to the homogeneity of the studies (I2 = 10%), the fixed effects model was used for the analysis. Based on the obtained results, the standardized mean difference was reported as 0.749 (CI 95% 0.555–0.943). The obtained result showed the positive effect of the procedure and the improvement of patients' pain after the surgery (Fig. 2**).**

### Myelopathy

**Fig. 3:**
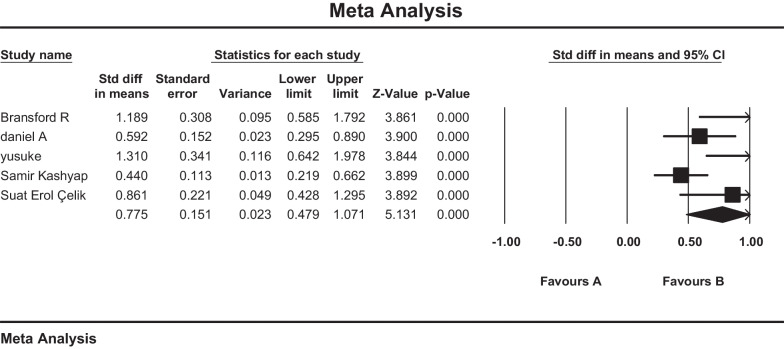
forest plot of standardized mean difference of Nurick grades

Myelopathy outcomes using Nurick score were reported in five studies. These studies examined a total of 199 cases. Random-effects model was used due to the heterogeneity of the studies (*I*^2^ = 64.4%). Based on the obtained results, the standardized mean difference was reported as 0.775 (CI 95% 0.479–1.071). The obtained result showed the positive effect of the procedure and improvement of patients' myelopathy after the operation (Fig. 3**).**

### Complications

Eight studies undergoing a transfacet pedicle-sparing approach assessed postoperative complications and neurological deterioration. There was no mortality or wrong-level surgery reported. The pooled overall complication was 12.4% (32/258) and 3.5% (9/258) neurological worsening as shown in **(**Table 4**)**. In the current analysis, four studies with a total of 166 patients reported an average hospital stay of 4.6 days [36–39]. Five studies with 182 patients reported an average blood loss of 580 cc. Follow-up time in these five studies was 13.8 months, and 5 patients were lost to follow-up [36–40].

**Table 4:** Studies that reported complications

Authors, Year	Neurological deterioration	Wound Infection	Pulmonary complication	Instrument migration/failure	Others
	1	4			1 large seoma
Arnold et al. [51]	1	1			
Nishimura et al. [40]		1			
Yang et al. [59]	3				
Carr et al. [37]		2	3	4	1 Epidural hematoma
Çelik et al. [54]					1Incomplete disc removal
Kashyap et al. [38]	4	1	1		3 recurrent disc herniation
Ovalioglu et al. [39]					1 Dural tear

## Discussion

### Historical perspective

Thoracic disc herniation was first described by Middleton and Teacher in 1991 [13], and since that time, management of TDH remains controversial [41]. Furthermore, the vagueness of clinical presentation, the low incidence rate of TDH pathology, the complexity of thoracic spine anatomy, and the availability of multiple surgical approaches make the management of thoracic disc herniation challenging [10]. Therefore, in perioperative decision-making, it is crucial to consider essential factors such as the location and texture of the herniated disc, the patient’s level of functionality, and the surgeon’s level of expertise [41]. Since the abandonment of laminectomy and fusion for the management of TDH due to high mortality, morbidity, and complication rate, many surgical approaches were utilized and improved patients' clinical neurological symptoms [28, 29, 41–45]. Some of these surgical approaches undertake extensive bone resection and tissue dissection which lead to disruption of the thoracic spine anatomy with the potential of high cardiopulmonary complications and axial back pain [27, 28, 46]. To decrease the occurrence of complications, minimally invasive posterolateral approaches, such as transpedicular, trasfacet pedicle-sparing, and endoscopic techniques, have become more popular [10, 41, 47, 48]. An increasing number of studies indicate posterolateral approaches achieves a similar outcome to the anterior transthoracic approach with a shorter hospital stay and lower complication rate [41, 45, 46]. Therefore, our aim is to assess the surgical outcome, complication rate, feasibility, and applicability of transfacet pedicle-sparing approach in patients undergoing thoracic discectomies.

### Patient demographics and characteristics

The presenting symptoms and neurological deficits experienced in our pooled studies were similar to those reported in literature undergoing other surgical approaches for thoracic disc herniation. In our study, the mean patient age was 50.19 ± 5.54 years and 51.5% were male and 48.5% were female **(**Table 1**).** The most common clinical presentation was myelopathy and radiculopathy. The most herniated disc was below the T6-7 segment **(**Table 2**)**. Historically multiple level herniation was thought to be rare [49, 50]; however, the revolution of imaging technology not only increased the incidence of TDH, but it also increased the frequency of multiple disc herniation in the thoracic vertebrae [8]. In our analysis, four studies reported a multilevel disc herniation and 38% (36/95) of patients had multilevel disc herniations [36, 37, 39, 51].

According to the literature, the anterior transthoracic approach is typically preferred for central and calcified discs [10, 52]. Furthermore, Stillerman et al. and colleagues proposed transfacet pedicle-sparing approach, which involves the preservation of the lateral facet. While this technique may present limitations in accessing central and calcified thoracic discs, preserving the lateral facet was thought to be critical in maintaining the stability of the thoracic spine and reducing axial back pain [53]. However, in our study, central discs accounted for 30% (52), and 18% (31) were calcified, out of the total 176 herniated discs **(**Table 3**)**. Notably, the authors in our analysis have modified Stillerman et al. surgical technique by removing the entire facet, which resulted in an improved surgical corridor with diagonal access to the disc space while minimizing dural retraction. Consequently, the authors used instrumentation and segmental fusion to address stability at the thoracic levels [36, 37, 39, 40, 54, 55].

### Overall pain and neurological improvement

Aggressive cord manipulation and the extent of bone removal and muscle dissection increase the likelihood of patients suffering from perioperative neurological deterioration and chronic back pain [53]. Crafoord et al. first described the anterior thoracic approach which remains to be the gold standard approach to achieve spinal cord decompression in calcified and giant central disc herniation [56]. However, due to rib resection, extensive muscle dissection, and pleural violation, it is subjected to postoperative cardiopulmonary complications [41, 53, 57]. Transfacet pedicle-sparing approach avoids the need for cord retraction and extensive muscle dissection decreasing the chance of neurological worsening and chronic back pain [41, 45, 46]. In this meta-analysis, both visual analog scale (VAS) and Nurick scores show a positive effect with the improvement of the patient's pain and neurological status. The standardized mean difference for VAS was 0.749 (CI 95% 0.555–0.943) across four studies using the fixed-effects model due to the homogeneity of the studies (*I*^*2*^ = 10%) [36, 37, 54, 58]. Neurological status improved in five studies using the Nurick score with a standardized mean difference of 0.775 (CI 95% 0.479–1.071) via a random-effects model due to heterogeneity (*I*^*2*^ = 64.4%) [36–38, 40, 54]. Our study demonstrates that transfacet pedicle-sparing approach is an acceptable technique in thoracic disc herniation.

### Overall complications

In the literature, the anterior approach was associated with higher overall postoperative complications [41, 46]. Hurley et al. in a systemic review compared the clinical outcomes of patients undergoing the anterior versus posterior approach for TDH. He reported that complication rates were 23% (193/842) for the anterior approach compared to 14% (43/314) in the posterior approach group [45]. Furthermore, Yoshihara et al. analysed 25,413 TDH cases from the National Inpatient Sample (NIS) and reported a complication rate of 26.8% (1529/5698) in the anterior approach compared to the nonanterior approach of 9.6% (1890/19715) [46]. Additionally, Kerezoudis et al. and colleagues investigated national registry surgical outcomes in 388 patients for anterior, lateral, and posterior approaches and reported a 27% (12/65) complication rate in the anterior approach [41]. In our study, eight studies reported a complication rate, of which the overall complication was 12.4% (32/258) and 3.5% (9/258) had neurological worsening (Table 4) [36–38, 40, 51, 54, 59].

Due to the higher complication rate in the anterior approach, it has been reported that the length of hospital stay (LOS) for the anterior approach was higher (median 6 days) and had significantly higher odds of having a pronged LOS (more than 7 days) compared to laminectomy, lateral and transpedicular approaches [41]. In the current analysis, four studies with a total of 166 patients reported an average hospital stay of 4.6 days [36–39]. Five studies with 182 patients reported an average blood loss of 580 cc. Follow-up time in these five studies was 13.8 months, and 5 patients were lost to follow-up [36–40]. Our result shows transfacet pedicle-sparing approach has a lower complication rate and length of hospital stay as compared to other surgical approaches that treat thoracic disc herniation.

## Segmental fusion and instrumentation

In the literature, the topic of whether to fuse or not after thoracic discectomy is a subject of debate, with proponents contending that fusion supports thoracic spine stability reducing postoperative axial back pain, while others contend that fusion and instrumentation introduce postoperative infections and future reoperation [10, 48]. Since the mechanical interactions between the vertebrae, intervertebral discs, rib cage, and sternum play a significant role in maintaining the thoracic spine stability, fusion is typically not advised for the thoracic spine if there is no pre-existing deformity or kyphosis unless there are giant calcified discs or multilevel discectomies [60]. In the anterior transthoracic approach where extensive bone resection is performed, studies have found an increasing number of fusions when compared to the posterior approach [46]. Furthermore, Quint et al. and colleagues reported 1.8% (3/167) postoperative instability after thoracoscopically treated discectomies, and they believe that fusion might help prevent the instability in the lower thoracic levels where the spine is less stiff and more flexible [48]. Additionally, Stillerman et al. recommend against fusion in transfacet pedicle-sparing approach when the lateral facet is preserved. Similar to existing literature, we found mixed opinions on fusion in our analysis. Four studies fused all the single-level herniations [36, 37, 40, 59], whereas one study performed fusion only when there were 3 consecutive level herniations [51]. 3 studies did not fuse their patients even after performing total facetectomies [38, 39, 54]. More studies with longer follow-up are needed to provide evidence if complete fasciectomy indeed leads to spine instability making fusion a requirement for transfacet pedicle-sparing approach.

### Strengths and limitations

The objective of the current study is to provide a comprehensive summary of the clinical outcomes associated with transfacet pedicle-sparing approach in thoracic disc herniations. This study aims to provide valuable insights into the judicious selection of minimally invasive surgical approaches with minimal complication rates for the appropriate patient selection. This study has multiple inherent limitations and biases. Search criteria were restricted to English articles published in the EMBASE, PubMed, and Cochrane Library databases. The meta-analysis is based on retrospective case series, which may have selection biases and may not represent the overall population. Furthermore, incomplete data collection and unrecorded important variables may lead to potential confounding factors that can affect the results. Apart from assessing motor scores, Nurick levels, ASIA scores, and VAS scores, no formal outcome instruments were used which can potentially introduce operator error depending on the surgeon's training level. Additionally, the absence of a control group makes it difficult to determine the actual impact of the intervention as there may be other factors responsible for the observed outcomes. Although this analysis comes from retrospective case series, the patients' symptomatic improvement without any concomitant morbidity confirms the procedure's efficacy and safety. Future studies should concentrate on the long-term follow-up of patients who underwent a transfacet approach for thoracic discectomy, specifically regarding late collapse, mechanical back pain, and fusion revision. Therefore, while our meta-analysis can provide valuable insights into clinical practice, it is important to acknowledge its limitations and interpret the findings with caution.

## Conclusion

This systemic review and meta-analysis evaluated pre-operative and post-operative variables reported in the literature by studies that performed transfacet pedicle-sparing approach to treat thoracic disc herniation. Of the variables that were assessed VAS and Nurick score were significantly associated with post-operative reduced pain and neurological improvement. The technique is associated with lower rates of complications and a shorter hospital stay compared to other surgical approaches. Our study provides evidence to support the use of the transfacet pedicle-sparing approach in the surgical management of thoracic disc herniation. The technique has been shown to be safe and effective, with several advantages over other surgical approaches for the right patient. This information can assist clinicians in making informed decisions when selecting the most appropriate surgical technique for their patients with thoracic disk herniation.

## Data Availability

All data generated or analysed during this study are included in this published article.
